# Monthly Summary

**Published:** 1887-11

**Authors:** 


### Monthly Summary.

---

TROPHIC NERVES.—The actual demonstration of the existence of trophic nerve fibres, apart from vaso-motor fibres, has not until recently been made, although many evidences pointing to that conclusion have been put forward. Dr. Joseph, of Berlin, in a number of experiments on cats, produce facts which go to prove the existence of trophic nerve fibres in the peripheral nerves. The complication of vascular changes induced by the removal of the vaso-motor fibres from a part must be excluded in order to prove definitely that any nutritive changes occurring after section of its nerve are due to the loss of trophic influence. These conditions were carefully considered, and in order to avoid vaso-motor changes, the second cervical nerve was selected, which, as will be seen, contains no vaso-motor fibres. This nerve was used in all experiments. Mere section of the trunk of the nerve produced no effect, as reunion rapidly took place, so that it became necessary to remove a considerable portion of the trunk to prevent this occurrence. In some of the animals the ganglion on the posterior nerve-root was also removed. The changes induced were limited to the area of distribution of the nerve, and in those where the ganglion was removed similar changes occurred in the cutaneous area of distribution of the fifth cranial nerve of the corresponding side, coming on in different animals at a period varying from five to twenty-seven days. The changes consisted of loss of hair, at first localized, afterwards gradually extending, situated above and behind the ear where the trunk was only severed: above the eye and over the cheek, where part of the trunk and the ganglion on the posterior root were removed. After a time complete baldness and a shiny atrophied state of the skin made their appearance. No increased vascularity, inflammation or any other change whatsoever could be discovered in the skin. The hair was examined for parasites, but none could be found. Microscopic examination of the skin failed to detect anything beyond atrophy of the hair follicies; there was no evidence of inflammatory or other changes. From these experiments, Dr. Joseph concludes that trophic nerve fibres exists apart from vaso-motor fibres, and entirely independent of them; and he explains the similar changes in the area of distribution of the fifth cranial nerve by assuming that it derives its trophic supply from the ganglion on the posterior root of the second cervical nerve from which fibres join the ascending root of the fifth.—*Virchows Archive*, Bd. cvii., Hft. 1.—*The Practitioner*, July, 1887.

---

DEATH DURING ETHER ANÆSTHESIA.—From our exchanges we have received accounts of a fatal case during the administration of ether. It occurred during the operation for hæmorrhoids in the person of a middle-aged man, under the care of Prof. D. Hayes Agnew, M. D., of Philadelphia. From the *Philadelphia Medical Times* of the 20th inst., we extract the following:

"The coroner's physician, Dr. Formad, made an autopsy, and discovered evidences of long-standing inflammatory and degenerative disease in the brain and medulla oblongata. The lungs were not engorged, but were collapsed. Death was probably due to the immediate shock of the operation or excessive apprehension. The lesions found were sufficient to cause death under any unusual excitement or emotion, but they do not correspond with the usual effects of ether when taken in a toxic dose."—*Southern Practitioner*.

DEATH OF A LADY AFTER HAVING SEVENTEEN TEETH EXTRACTED.—On Saturday, June 11th, Mrs. William E. Berry, living near Sparta, Hancock county, Georgia, had seventeen teeth extracted. She retired in the evening, feeling badly and nervous, and during the night died. She was a young married lady. Her death was unaccounted for. It is thought by some that the extraction of the teeth was the cause of her death. The extraction was done by Dr. Buck, who travels from place to place extracting teeth without pain by use of a preparation he calls Electro-dentos. The anæsthesia is affected by applying the preparation to the beaks of the forceps. Dr. Buck is not a practicing dentist, but devotes his time to extracting teeth and selling rights to dentists. He claims that it relieves pain entirely.

Now, as to whether the death of Mrs. Berry was caused from the effects of his preparation, or was simply produced by the nervous shock, or some other condition of her system, is a matter of conjecture. We record the facts as they came to us.—*Dental Luminary.*

---

EFFECTS OF STUDY ON THE TEETH.—Among the hard-worked pupils of the Paris public schools, the teeth become deteriorated in a few months after entry. The second dentition is often premature. These observations confirm the statements of Dr. J. L. Williams, who has given great attention to this subject. He has shown that any mental strain shows itself upon the teeth in a short time, both in increased decay as well as in increased sensibility of dentine. Dr. D. M. Parker has reported that these same changes are always apparent in men who are training for athletic trials.—*Boston Medical Journal.*

---

A DENTIST'S BILL.—Three teeth in the head of G. Onesti were operated upon recently by Dr. W. J. Younger, who sent in his bill for $336 50, alleged to be due him as an aggregate for twenty-two hours and twenty minutes' work at $15 an hour. Onesti refused to settle, claiming that he considered $70 to be a reasonable charge. Suit was brought by the dentist, and the jury awarded the plaintiff the $70 offered him at first.—*San Francisco Examiner.*

